# Metaplastic breast cancer with a unique presentation and complete response to chemotherapy: a case report

**DOI:** 10.1186/s12905-024-03134-8

**Published:** 2024-05-11

**Authors:** Fouad Nahhat, Modar Doyya, Kareem Zabad, Hazem Ksiri

**Affiliations:** 1 Faculty of Medicine, Damascus University, Damascus, Syria; 2 Department of Oncology, Albairouni University Hospital, Damascus, Syria

**Keywords:** Metaplastic breast Cancer, Spindle cell carcinoma, Triple-negative breast Cancer, Axillary Lymph Node, Chemotherapy, Doxorubicin, Cyclophosphamide

## Abstract

**Background:**

Metaplastic breast carcinomas are a rare variant group of breast carcinomas. They are usually high-grade and triple-negative tumors. They often present with large primary tumor sizes. However, the involvement of axillary lymph nodes is infrequent at the time of diagnosis. Metaplastic breast carcinomas are associated with a worse prognosis and a poorer response to chemotherapy in comparison with other non-metaplastic triple-negative breast cancers. Up until this point, there are no specific treatment recommendations for metaplastic breast carcinomas beyond those intended for invasive breast cancer in general.

**Case presentation:**

A 40-year-old woman complained of a palpable mass in her left axilla. On ultrasonography, the mass was solid, spindle-shaped, hypoechoic with regular borders, and exhibited decreased vascularity. At first, the mass appeared to be of a muscular origin. There was not any clinical nor ultrasonic evidence of a primary breast tumor. On magnetic resonance imaging, the axillary mass was a well-defined with regular borders, measuring 24 × 35 mm. Needle biopsy showed a spindle cell tumor with mild to moderate atypia. The subsequent surgical resection revealed a spindle cell neoplasm within a lymph node, favoring a metastatic origin of the tumor. The tumor cells lacked expression of estrogen, progesterone, and HER2 receptors. PET-CT scan indicated pathological uptake in the left breast. Accordingly, the patient was diagnosed with metaplastic breast cancer that had metastasized to the axillary lymph node. She commenced a combined chemotherapy regimen of doxorubicin and cyclophosphamide. After six treatment cycles, she underwent left modified radical mastectomy with axillary lymph node dissection. Pathological examination of the specimens revealed a total burn-out tumor in the breast due to excellent treatment response. There were no residual tumor cells. All dissected lymph nodes were free of tumor. At the one-year follow-up, the patient showed no signs of tumor recurrence.

**Conclusion:**

This report sheds light on a distinctive presentation of metaplastic breast carcinoma, emphasizing the need for vigilance in diagnosing this rare and aggressive breast cancer variant. In addition, the patient’s remarkable response to chemotherapy highlights potential treatment avenues for metaplastic breast cancer.

## Background

Metaplastic breast carcinoma (MpBC) makes up an estimated 0.2–5% of all invasive breast cancers. It represents a heterogeneous group of tumors in which neoplastic cells display differentiation towards squamous epithelium as well as mesenchymal components, such as spindle, chondroid, osseous, or rhabdoid cells [1]. 

The WHO classification of metaplastic breast cancer subtypes includes mixed metaplastic carcinoma, low-grade adenosquamous carcinoma, fibromatosis-like, squamous cell carcinoma, spindle cell carcinoma, myoepithelial carcinoma, and metaplastic carcinoma with mesenchymal differentiation (chondroid, osseous) [1].

MpBCs are often aggressive in nature, typically presenting as high-grade tumors with a triple-negative phenotype (negative for estrogen receptor, progesterone receptor, and HER2) [2]. 

Upon presentation, most MpBC patients tend to exhibit large primary tumor sizes, often exceeding 5 cm. Nevertheless, the involvement of axillary lymph nodes is infrequent at the time of diagnosis compared to other types of triple-negative breast cancer (TNBC) [3]. 

Considering that MpBC possesses a worse prognosis than other non-metaplastic TNBCs, carries twice the risk of recurrence, and has a shorter disease-free and overall survival (71% 5-year overall survival for MpBC compared to 88% for IDC) [4], it is worth noting that The National Comprehensive Cancer Network (NCCN) guidelines do not offer specific treatment recommendations for MpBC beyond those intended for invasive breast carcinoma in general [5].

Over the past years, conventional therapies including surgery, chemotherapy, and radiation have been used to treat MpBC. However, the inadequacy of these treatment methods, as reflected by poor survival rates, has necessitated the need for novel therapeutic options such as targeted and immunotherapies, which are still undergoing experimental trials and evaluation [6]. 

The objective of this study was to report a case of metaplastic breast cancer with a unique presentation and complete response to chemotherapy.

## Case presentation

A 40-year-old woman presented to the clinic complaining of a palpable mass in her left axilla, which had been present for two months. Otherwise, she had no other complaints. Her medical history was unremarkable.

Upon physical examination, the left axillary mass was fixed and measured approximately 30 mm in size, displaying no visible signs on the skin such as redness, ulceration, or swelling. Additionally, there was nipple induration noted in both breasts in the absence of any palpable masses.

**Fig. 1:**
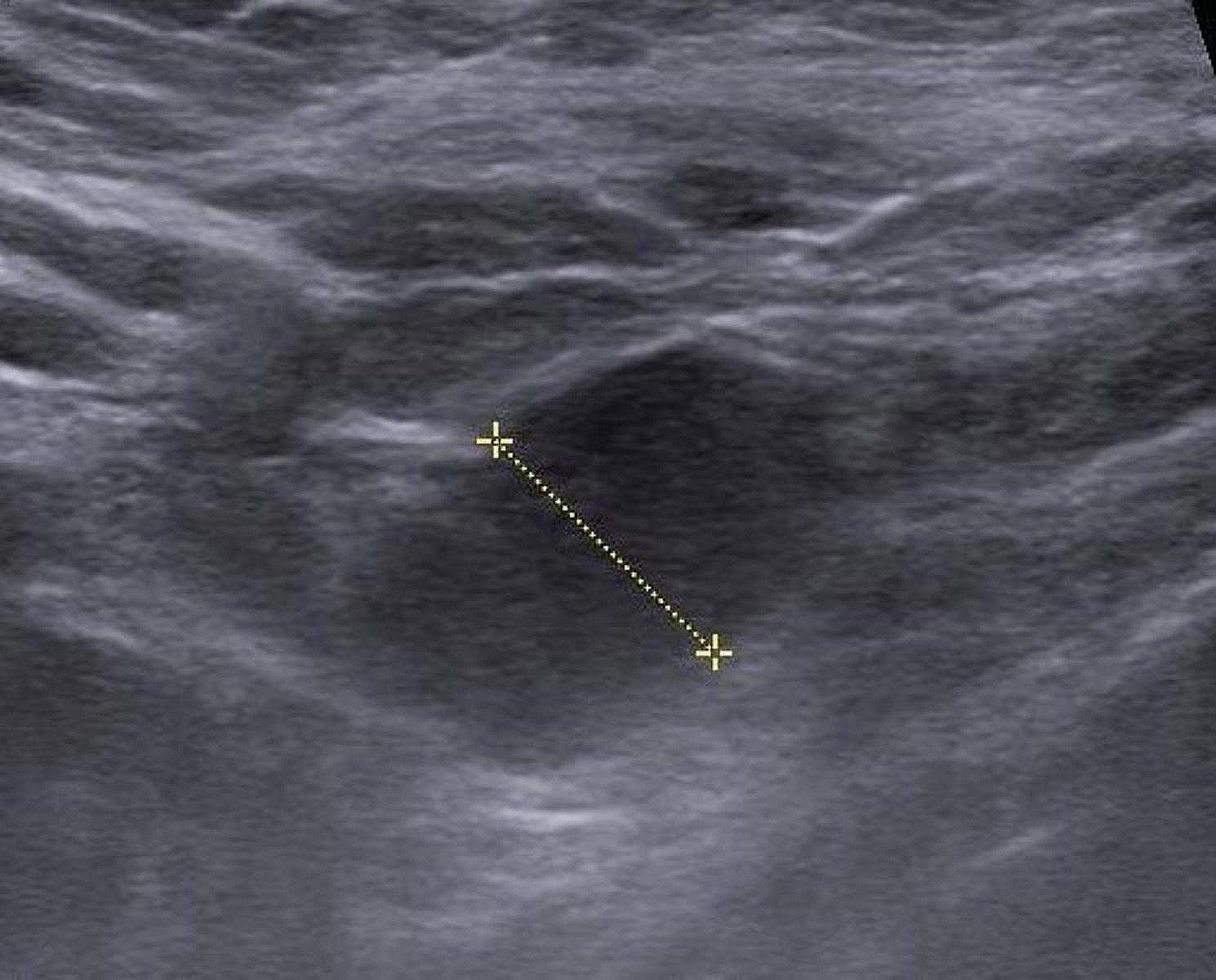
Ultrasonography of the axillary mass

Ultrasonography revealed numerous benign cysts in both the left and right breasts. In addition, it detected two fibroadenomas in the left breast: one measuring 13–20 mm and the other, smaller at 12 mm but notably hypoechoic. The left axillary mass was solid, spindle-shaped, hypoechoic with regular borders, and exhibited decreased vascularity (Fig. 1). It measured approximately 31 mm. It was adjacent to the axillary artery and contiguous with the tendon of one of the rotator cuff muscles. Thus far, the mass appeared to be of a muscular origin. Moreover, ultrasonography identified three enlarged lymph nodes that were hypoechoic and measuring 7–8 mm.

Mammography did not reveal any additional findings beyond those obtained by ultrasound.

**Fig. 2:**
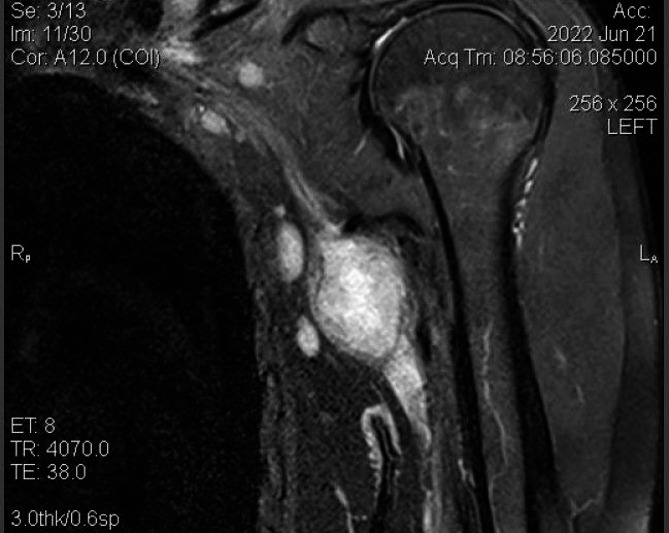
Magnetic resonance imaging (MRI) of the axillary mass

Magnetic resonance imaging (MRI) of the left axilla demonstrated a well-defined mass with regular borders, measuring 24 × 35 mm. It was directly adjacent to the axillary nerve that they were practically indistinguishable (Fig. 2). On T1-weighted images, the lesion exhibited low signal intensity, while on T2-weighted images, it demonstrated heterogeneously high signal intensity. Following contrast administration, the mass showed heterogeneously marked enhancement.

In accordance with MRI findings, the differential diagnosis primarily included nerve tumors such as schwannoma and possibly a metastatic lymph node.

A needle biopsy of the axillary mass showed a spindle cell tumor with mild to moderate atypia. An excisional biopsy was recommended for comprehensive evaluation.

The subsequent mass resection pathology revealed a malignant high-grade spindle cell neoplasm within a lymph node, favoring a metastatic origin of the tumor. Immunostaining of the sample was positive for CK (Cytokeratin), Vimentin (focally), and S100 markers and negative for TLE (Transducin-like enhancer of split), CD34, CK5/6, and p63 markers. Furthermore, the tumor cells lacked expression of estrogen, progesterone, and HER2 receptors (triple negative). The Ki-67 index was low (< 10%).

**Fig. 3:**
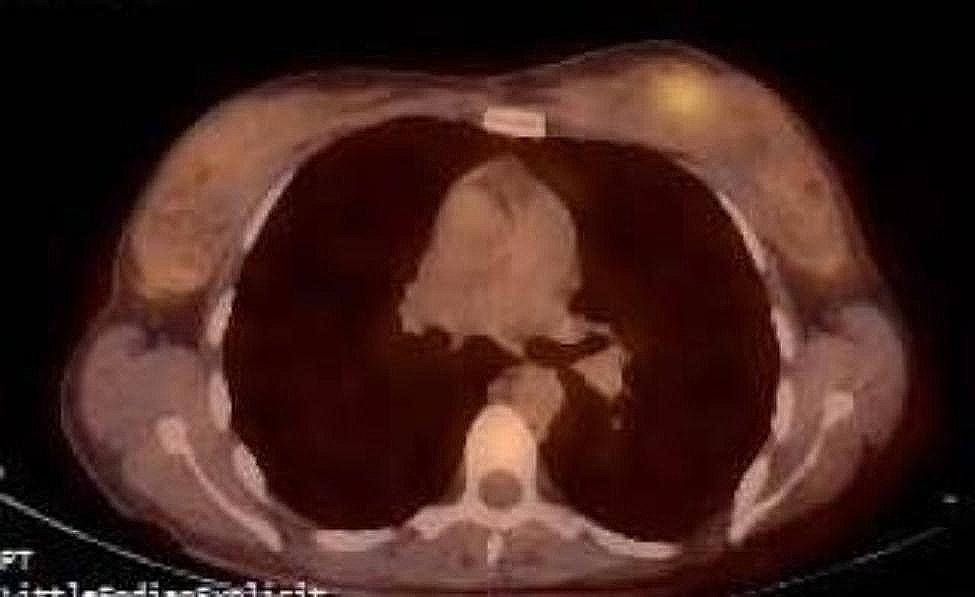
PET-CT scan showing pathological uptake of FDG in the left breast

PET-CT scan indicated pathological uptake of FDG in the upper inner quadrant of the left breast (SUV = 3) (Fig. 3). There was no pathological metabolic activity observed in other parts of the body.

Accordingly, the diagnosis was triple-negative metaplastic breast cancer (of a spindle cell carcinoma subtype) that had metastasized to the axillary lymph node.

The patient subsequently commenced a combined chemotherapy regimen of doxorubicin (Adriamycin) and cyclophosphamide (AC).

After completing six treatment cycles, there was a mild decrease in nipple induration and the patient underwent left modified radical mastectomy with axillary lymph node dissection as the accurate primary tumor location could not be identified because no biopsy was taken from the primary tumor and no clip was placed in its site, as this technique is not available in Syria anyway [7, 8]. 

Pathological examination of the surgical specimens revealed a total burn-out tumor in the breast due to excellent treatment response. There were no residual tumor cells. All 19 dissected lymph nodes were reactive and free of tumor.

At the one-year follow-up, the patient showed no signs of tumor recurrence.

## Discussion and conclusions

Metaplastic breast carcinoma (MpBC) is a rare subtype of invasive breast cancer characterized by the presence of both epithelial and mesenchymal elements and typically presents as an aggressive triple-negative tumor [1, 2]. 

MpBC usually manifests as a palpable breast mass [9]. However, it often spares axillary lymph nodes. In an analysis of 892 MpBC cases, only 174 (21.9%) had axillary node involvement at presentation [2]. Even in studies with smaller samples, the percentage of patients with positive axillary lymph nodes at diagnosis did not exceed 38–40% [10, 11]. 

In contrast, our patient presented uniquely with a relatively big axillary mass, which was later identified as a metastatic lymph node in the absence of any clinical evidence of the primary breast tumor.

Ultrasonography of MpBCs usually shows irregular tumor shadow, micro-lobulated margins, complex echogenicity, parallel orientation, and posterior acoustic enhancement [9]. On the other hand, the most common feature of MpBCs on MRI is the high intensity of signal on T2-weighted images [12]. In addition, these tumors usually have irregular margins and display heterogeneous internal enhancement on MRI [9].

In our case, ultrasonography and mammography of the breasts were only remarkable for multiple bilateral cysts and two benign lesions in the left breast, which were considered fibroadenomas at that time. They did not reveal any suspicious findings, which made the breast origin of the axillary mass less likely at first.

However, MpBCs tend to display more benign features, such as round or oval shapes with well-defined borders, in comparison with ductal carcinomas [13]. The circumscribed margins of the mass on mammography might reflect the spindle-cell component of these tumors [14].

Relative to other non-metaplastic TNBCs, MpBCs have a worse prognosis and poorer response to chemotherapy. While TNBCs typically exhibit a pathological complete response (pCR) rate of 33.6% to neoadjuvant chemotherapy [15], the response rates for MpBCs are generally lower. Results from four studies on neoadjuvant chemotherapy for MpBC indicated pCR rates of 2% in one study involving 44 patients [16], 17% in another study with 29 patients [17], 11% in a study with 18 patients [18], and 16% in the last one involving six patients [19].

Despite the unique presentation, our patient achieved a pCR to the combined chemotherapy regimen (AC), highlighting its potential role in unusual cases of MpBC.

In the upcoming era, chemotherapy alone might not constitute the definitive curative approach for MpBC. However, it could be used in combination with targeted and immunotherapies, which are still undergoing experimental trials and evaluation [6].

Novel therapeutic options for MpBC are emerging based on the specific molecular characteristics of the disease. TP53 mutations, which are very common in MpBC, and mutations in PI3K have been identified as promising targets for therapy [20–22].

TP53 mutations are also associated with elevated VEGF-A levels, suggesting potential sensitivity to anti-VEGF agents such as bevacizumab [23, 24]. Notably, mTOR inhibitors such as temsirolimus and everolimus have shown secondary effects on angiogenesis, providing the base for investigating combinations with bevacizumab in clinical trials [25, 26]. Furthermore, the combination of temsirolimus with bevacizumab and various chemotherapy agents, including platinums, taxanes, and anthracyclines, has been studied [27].

Additionally, MpBC is characterized by the overexpression of PD-L1 and high tumor-infiltrating lymphocytes (TIL), which set the basis for treating MpBC with immunotherapeutic agents [22, 28]. 

In summary, this report sheds light on a distinctive presentation of metaplastic breast carcinoma (MpBC), emphasizing the need for vigilance in diagnosing this rare and aggressive breast cancer variant. In addition, the patient’s remarkable response to chemotherapy highlights potential treatment avenues for MpBC.

One limitation of our study was the absence of suspicious findings on breast imaging, which precluded the opportunity to perform a biopsy for confirmation. Consequently, the diagnosis relied on the correlation between PET-CT scan findings and lymph node pathology.

Another limitation to acknowledge is that our study was conducted on a single patient. While this case provided valuable insights, the findings may not be fully generalizable to a broader population and larger studies are required.

## Data Availability

All data are included in this article.

## Abbreviations

MpBC: Metaplastic Breast Carcinoma
WHO: World Health Organization
HER2: Human Epidermal Growth Factor Receptor 2
TNBC: Triple-negative Breast Cancer
OS: Overall Survival
NCCN: The National Comprehensive Cancer Network
AC: Adriamycin (Doxorubicin) & Cyclophosphamide
MRI: Magnetic Resonance Imaging
CK: Cytokeratin
TLE: Transducin-like enhancer of split
PET: Positron Emission Tomography
FDG: F-18 fluorodeoxyglucose
pCR: Pathological Complete Response
TP53: Tumor Protein 53 gene
PI3K: Phosphoinositide 3-kinases
VEGF-A: Vascular Endothelial Growth Factor A
mTOR: Mammalian Target of Rapamycin
PD-L1: Programmed Death-ligand 1
TIL: Tumor-infiltrating Lymphocytes

## References

[1] Reis-Filho JS (2019). Metaplastic carcinoma. The WHO classification of tumours breast tumours.

[2] Pezzi CM (2007). Characteristics and treatment of metaplastic breast cancer: analysis of 892 cases from the National Cancer Data Base. Ann Surg Oncol.

[3] Yang X, Tang T, Zhou T (2022). Prognosis and clinicopathological characteristics of metaplastic breast cancer: a meta-analysis. Medicine.

[4] Nelson RA, Guye ML, Luu T, Lai LL (2015). Survival outcomes of metaplastic breast cancer patients: results from a US population-based analysis. Ann Surg Oncol.

[5] NCCN Clinical Practice Guidelines in. Oncology (NCCN Guidelines®)—Breast Cancer. https://www.nccn.org. 2021 (version 1. 2021).

[6] Tray N, Taff J, Adams S (2019). Therapeutic landscape of metaplastic breast cancer. Cancer Treat Rev.

[7] Nahhat F, Doyya M, Zabad K, Laban TA, Najjar H, Saifo M, Badin F (2023). Breast cancer quality of care in Syria: screening, diagnosis, and staging. BMC Cancer.

[8] Nahhat F, Badin FB, Saifo M (2023). How does the war affect breast cancer quality of care? The Syrian experience. J Clin Oncol.

[9] Choi BB, Shu KS (2012). Metaplastic carcinoma of the breast: multimodality imaging and histopathologic assessment. Acta Radiol.

[10] Gibson GR, Qian D, Ku JK, Lai LL (2005). Metaplastic breast Cancer: clinical features and outcomes. Am Surg.

[11] Park JM, Han WK, Moon WK (2000). Metaplastic carcinoma of the breast: mammographic and sonographic findings. J Clin Ultrasound.

[12] Velasco M, Santamaría G, Ganau S, Farrús B, Zanón G, Romagosa C, Fernández PL (2005). MRI of metaplastic carcinoma of the breast. AJR. Am J Roentgenol.

[13] Yang WT, Hennessy B, Broglio K (2007). Imaging difference in metaplastic and invasive ductal carcinoma of the breast. Am J Roentgenol.

[14] Petterson SK, Tworek JA, Roubidoux MA (1997). Metaplastic carcinoma of the breast: mammographic appearance with pathologic correlation. Am J Roentgenol.

[15] Cortazar P (2014). Pathological complete response and long-term clinical benefit in breast cancer: the CTNeoBC pooled analysis. Lancet.

[16] Wong W, Brogi E, Reis-Filho JS (2021). Poor response to neoadjuvant chemotherapy in metaplastic breast carcinoma. npj Breast Cancer.

[17] Han M (2019). Metaplastic breast carcinoma: a clinical-pathologic study of 97 cases with subset analysis of response to neoadjuvant chemotherapy. Mod Pathol.

[18] Al-Hilli Z (2019). Metaplastic breast cancer has a poor response to neoadjuvant systemic therapy. Breast Cancer Res Treat.

[19] Cimino-Mathews A (2016). A clinicopathologic analysis of 45 patients with metaplastic breast carcinoma. Am J Clin Pathol.

[20] Ng CKY, Piscuoglio S, Geyer FC (2017). The landscape of somatic genetic alterations in metaplastic breast carcinomas. Clin Cancer Res.

[21] Piscuoglio S, Ng CKY, Geyer FC (2017). Genomic and transcriptomic heterogeneity in metaplastic carcinomas of the breast. npj Breast Cancer.

[22] Tray N, Taff J, Singh B (2018). Metaplastic breast cancers: genomic profiling, mutational burden and tumor-infiltrating lymphocytes. Breast.

[23] Wheler JJ, Janku F, Naing A (2016). TP53 alterations correlate with response to VEGF/VEGFR inhibitors: implications for targeted therapeutics. Mol Cancer Ther.

[24] Schwaederle M, Lazar V, Validire P (2015). VEGF-A expression correlates with TP53 mutations in non-small cell lung cancer: implications for antiangiogenesis therapy. Cancer Res.

[25] Tzanninis IG, Kotteas EA, Ntanasis-Stathopoulos I, Kontogianni P, Fotopoulos G (2016). Management and outcomes in metaplastic breast cancer. Clin Breast Cancer.

[26] Moroney J, Fu S, Moulder S (2012). Phase I study of the antiangiogenic antibody bevacizumab and the mTOR/hypoxia-inducible factor inhibitor temsirolimus combined with liposomal doxorubicin: tolerance and biological activity. Clin Cancer Res.

[27] Basho RK, Yam C, Gilcrease M et al. Comparative effectiveness of an mTOR-Based systemic therapy regimen in advanced, metaplastic and nonmetaplastic triple-negative breast cancer. Oncologist. 2018. 10.1634/theoncologist.2017-0498 PMC6291334 30139837

[28] Joneja U, Vranic S, Swensen J (2017). Comprehensive profiling of metaplastic breast carcinomas reveals frequent overexpression of programmed death-ligand 1. J Clin Pathol.

